# Clinical Outcomes of Perioperative Immunotherapy in Resectable Non–Small Cell Lung Cancer

**DOI:** 10.1001/jamanetworkopen.2025.17953

**Published:** 2025-06-30

**Authors:** Aakash Desai, Karen Schwed, Laurynas Kalesinskas, Qianyu Yuan, Jonathan Bryan, Catherine Keane, Erin Fidyk, Emily Castellanos, Aaron B. Cohen, Katherine Harrison, George Ho, Anca Marinescu, Ticiana A. Leal

**Affiliations:** 1 Division of Hematology and Oncology, Department of Medicine, University of Alabama at Birmingham, Birmingham; 2 Flatiron Health, New York, New York; 3 Department of Radiation Oncology, Memorial Sloan Kettering Cancer Center, New York, New York; 4 Department of Hematology/Oncology, Fox Chase Cancer Center, Philadelphia, Pennsylvania; 5 Department of Medicine, Grossman School of Medicine, New York University, New York; 6 Department of Hematology and Medical Oncology, Winship Cancer Institute of Emory University, Atlanta, Georgia

## Abstract

**Importance:**

Lung cancer remains a leading cause of cancer-related mortality, with early-stage non–small cell lung cancer (NSCLC) accounting for 40% to 45% of new diagnoses. Despite the adoption of perioperative chemoimmunotherapy, clinical data on its use and outcomes remain limited.

**Objective:**

To evaluate clinical practice patterns and outcomes associated with neoadjuvant and adjuvant chemoimmunotherapy use in patients with resectable stage II to IIIA NSCLC.

**Design, Setting, and Participants:**

This retrospective cohort study obtained data from the Flatiron Health database containing deidentified, patient-level, electronic health record–derived data from approximately 280 cancer clinics in the US. The cohort included adults diagnosed with stage II to IIIA NSCLC between January 1, 2020, and October 31, 2023, who underwent surgical resection. Included patients were receiving neoadjuvant chemoimmunotherapy (nivolumab or pembrolizumab with platinum-based doublet chemotherapy) or adjuvant chemoimmunotherapy (atezolizumab or pembrolizumab with or without chemotherapy) after the US Food and Drug Administration approval of these therapies.

**Exposures:**

Neoadjuvant and adjuvant chemoimmunotherapy for resectable NSCLC.

**Main Outcomes and Measures:**

The primary outcome was clinical distant metastasis–free survival (DMFS), defined as time from treatment initiation to distant metastasis or death. Secondary outcomes included biomarker testing rates, time to treatment, and metastatic patterns.

**Results:**

Included in the analysis were 1334 patients with resectable stage II to IIIA NSCLC treated with immunotherapy, of whom 424 (median [IQR] age, 68 [63.0-74.0] years; 225 males [53.1%]) received chemoimmunotherapy with platinum-based doublet in the neoadjuvant setting and 910 (median [IQR] age, 69 [63.0-74.0] years; 441 males [48.5%]) received chemoimmunotherapy or immunotherapy alone in the adjuvant setting. The clinical DMFS at 18 months was 80.2% (95% CI, 75.0%-85.7%) for the neoadjuvant cohort and 83.0% (95% CI, 80.0%-86.0%) for the adjuvant cohort. Biomarker testing rates were higher in the adjuvant cohort compared to the neoadjuvant cohort (eg, programmed cell death ligand 1 testing: 72.2% vs 52.6%, *P* < .001). Common metastatic sites included brain, bone, and pleura, consistent across cohorts. Chemoimmunotherapy among patients who received surgery increased from 2022 to 2023 but remained under 30% of eligible patients (neoadjuvant setting: from 8.4% [82 of 966] to 13.8% [254 of 1842]; adjuvant setting: from 19.7% [372 of 1887] to 22.6% [401 of 1776]).

**Conclusions and Relevance:**

This retrospective cohort study found that chemoimmunotherapy was associated with favorable clinical DMFS outcomes in patients with resectable NSCLC. The findings highlight the need to address barriers to broader adoption of chemoimmunotherapy.

## Key Points

**Question:**

What are the clinical outcomes and use patterns of neoadjuvant and adjuvant chemoimmunotherapy in patients with resectable stage II to IIIA non–small cell lung cancer (NSCLC)?

**Findings:**

In this cohort study of 1334 patients with stage II to IIIA disease, the clinical distant metastasis–free survival (DMFS) at 18 months for patients in the neoadjuvant and adjuvant treatment cohorts indicated favorable DMFS outcomes.

**Meaning:**

The findings suggest that chemoimmunotherapy is associated with favorable clinical outcomes in resectable NSCLC; therefore, there is a critical need to address barriers to broader adoption of chemoimmunotherapy.

## Teaser

This cohort study evaluates the adoption and survival outcomes of US Food and Drug Administration–approved chemoimmunotherapies in the neoadjuvant or adjuvant settings for adults with stage II to IIIA non–small cell lung cancer (NSCLC).

## Introduction

Lung cancer remains one of the most common new cancer diagnoses, with approximately 240 000 cases estimated in 2023.^1^ Of these, early-stage (stage I-III) non–small cell lung cancer (NSCLC) accounts for about 40% to 45%, with the expected increase in prevalence given the increased adoption of recommended screening practices.^2^ In this setting, despite the use of platinum-based chemotherapy regimens, cancer recurrence and cancer-related mortality pose a major problem.^3^ Thus, there has been an increasing interest in incorporation of immunotherapy and targeted therapy in the perioperative setting to improve outcomes for patients with early-stage NSCLC.

Various approaches incorporating immunotherapy in the neoadjuvant, adjuvant, and (more recently) perioperative settings have been studied, with addition of immunotherapy being associated with improvement in outcomes over adjuvant platinum-based doublet chemotherapy alone, leading to many US Food and Drug Administration (FDA) approvals.^4^ Neoadjuvant nivolumab plus chemotherapy is approved for patients with resectable stage IB to IIIA NSCLC based on improvements in pathological complete response and event-free survival (EFS) (31.6 vs 20.8 months; hazard ratio [HR], 0.63; *P* = .005).^5^ Immunotherapy (pembrolizumab and atezolizumab) has also been approved in the adjuvant setting after adjuvant cytotoxic chemotherapy based on improvement in disease-free survival for patients with stage IB to IIIA NSCLC.^6,7^ More recently, perioperative pembrolizumab with platinum-containing chemotherapy as neoadjuvant treatment, and with continuation of single-agent pembrolizumab as adjuvant treatment, was approved for resectable (tumors ≥4 cm or node-positive) NSCLC.^8^ This approval was based on the KEYNOTE 671 (Efficacy and Safety of Pembrolizumab [MK-3475] With Platinum Doublet Chemotherapy as Neoadjuvant/Adjuvant Therapy for Participants With Resectable Stage II, IIIA, and Resectable IIIB [T3-4N2] Non-Small Cell Lung Cancer) trial, which demonstrated improvement in the median EFS and median overall survival after pembrolizumab plus platinum-based chemotherapy compared with placebo.^8^ Similarly, perioperative durvalumab with platinum-containing chemotherapy was also approved based on data from the AEGEAN (A Study of Neoadjuvant/Adjuvant Durvalumab for the Treatment of Patients With Resectable Non-small Cell Lung Cancer) study associating this treatment with improvement in median EFS.^9^

Although incorporation of immunotherapy in early-stage NSCLC has now become standard practice and is supported by guidelines, little is known about its use and outcomes in the clinical setting. Here, we leveraged a nationwide US oncology database to evaluate clinical practice patterns and outcomes associated with neoadjuvant and adjuvant chemoimmunotherapy use in patients with resectable stage II to IIIA NSCLC.

## Methods

This retrospective cohort study used the longitudinal Flatiron Health database, which included deidentified, patient-level structured and unstructured data on 280 000 patients with NSCLC. The data, curated via machine learning and expert human abstraction, originated from the electronic health record (EHR) of approximately 280 cancer clinics (approximately 800 sites of care) throughout the US.^10^ The WCG Institutional Review Board approved the study and waived the informed consent requirement because deidentified data were used. The study adhered to the Reporting of Studies Conducted Using Observational Routinely Collected Data (RECORD) reporting guideline, which extends the Strengthening the Reporting of Observational Studies in Epidemiology (STROBE) reporting guideline.

The present study cohort included adult patients (aged ≥18 years) with a new diagnosis of early-stage NSCLC (resectable stage II-IIIA using the eighth edition of the American Joint Committee on Cancer/Union for International Cancer Control tumor-node-metastasis system) between January 1, 2020, and October 31, 2023, and those who received surgery on or after January 1, 2021. The data cutoff was May 31, 2024. Patients were categorized based on receipt of neoadjuvant (chemoimmunotherapy prior to surgery) and/or adjuvant (chemoimmunotherapy within 12 weeks after surgery) therapy. Chemoimmunotherapy in both settings was limited to administration after the FDA approval in 2022 for the neoadjuvant cohort and in 2021 for the adjuvant cohort. Chemoimmunotherapy was limited to administration after the FDA approval, which was after July 2022 for the neoadjuvant cohort (accounting for surgical window) and after December 2021 for the adjuvant cohort. A sensitivity analysis of clinical distant metastasis–free survival (DMFS) was conducted among patients in the neoadjuvant chemoimmunotherapy cohort who later received adjuvant immunotherapy or who later did not receive adjuvant immunotherapy.

We described patient demographics (age, sex, race and ethnicity, and smoking status), Eastern Cooperative Oncology Group Performance Status, tumor histologic type, disease stage, programmed cell death ligand 1 (PD-L1) expression, and biomarker positivity. Clinical characteristics were extracted via machine learning models.^11^ Race and ethnicity were abstracted from the EHR and were self-reported by patients at the point of care. Categories included Asian, Black or African American, Hispanic or Latino, White, and other (American Indian or Alaska Native, Native Hawaiian or Other Pacific Islander, multiracial, or not otherwise specified). These classifications were used for descriptive purposes only.

The primary end point was clinical DMFS defined as time from index date (treatment start time) to date of metastases or death, with censoring based on last follow-up or data cutoff (May 2024).^12^ Death was defined through a composite mortality measure (formulated by Flatiron Health) that integrates structured and unstructured EHR data, which were then cross-referenced with a commercially available mortality database and the Social Security Death Index.^13^ The secondary end points included biomarker testing rates, time to treatment, and metastatic patterns.

Among patients with a diagnosis of NSCLC, only patients who had undergone surgical resection and were diagnosed with stage II to IIIA were included in the analysis. Patients diagnosed with stage 1B NSCLC were excluded from the analysis, as these patients were likely to forgo neoadjuvant chemotherapy and proceed with surgery alone. The neoadjuvant chemoimmunotherapy cohort included patients treated with nivolumab (FDA approval on April 4, 2022) or pembrolizumab (FDA approval on October 16, 2023) with platinum-based doublet therapy (cisplatin or carboplatin with another chemotherapy, including etoposide, vinorelbine, pemetrexed, gemcitabine, docetaxel, or paclitaxel) before surgery. Patients who did not undergo surgical resection after neoadjuvant immunotherapy were not included in the analysis. Patients in the adjuvant chemoimmunotherapy cohort received platinum-based doublet chemotherapy (cisplatin or carboplatin with another chemotherapy including etoposide, vinorelbine, pemetrexed, gemcitabine, docetaxel, or paclitaxel) and subsequent immunotherapy using either atezolizumab (FDA approval on October 15, 2021) or pembrolizumab (FDA approval on January 26, 2023) or immunotherapy alone (atezolizumab or pembrolizumab) within 84 days after surgery but received no neoadjuvant therapy.

### Statistical Analysis

Patient, cancer, and treatment characteristics of the neoadjuvant and adjuvant groups were summarized and compared using unpaired, 2-tailed *t* test (for continuous variables) or Pearson χ^2^ test (for categorical variables). Analysis of clinical DMFS for both cohorts was conducted using the Kaplan-Meier method. The HRs within treatment groups were evaluated using Cox proportional hazards regression models adjusted for age, race and ethnicity, sex, practice type, smoking status, disease stage, PD-L1 status, and histologic type. For the neoadjuvant cohort, time from initial diagnosis to neoadjuvant treatment initiation and time from neoadjuvant treatment initiation to surgery were obtained. Information was obtained on biomarker testing in the form of PD-L1 and actionable genomic alterations (*EGFR, ALK, KRAS, BRAF, MET, RET,* and *ROS1*).

Statistical significance was defined as a 2-sided *P* < .05. Data analysis was performed in July 2024 using R, version 4.1.3 (R Project for Statistical Computing).

## Results

Among 279 181 patients with a diagnosis of NSCLC, only 5748 who had undergone surgical resection and were diagnosed with stage II to IIIA disease met the inclusion criteria for the analysis. A total of 1334 patients received chemoimmunotherapy during their treatment timeline, of whom 424 (31.8%) received chemoimmunotherapy with platinum-based doublet in the neoadjuvant setting and 910 (68.2%) received platinum-based doublet with immunotherapy or immunotherapy alone in the adjuvant setting within 84 days after surgery. Baseline characteristics for both cohorts are described in the Table. The median (IQR) age was 68 (63.0-74.0) years for the neoadjuvant cohort and 69 (63.0-74.0) years for the adjuvant cohort, with no significant difference in age distribution (*P* = .23). Sex distribution was nearly balanced across both cohorts, with 199 females (47.0%) and 225 males (53.0%) in the neoadjuvant cohort and 469 females (51.5%) and 441 males (48.5%) in the adjuvant cohort (*P* = .13).

**Table.** Patient Demographic and Clinical Characteristics

Characteristic	Patients, No. (%)		*P* value
	Neoadjuvant cohort (n = 424)	Adjuvant cohort (n = 910)	
Age, median (IQR), y	68.0 (63.0-74.0)	69.0 (63.0-74.0)	.23
Sex			
Female	199 (46.9)	469 (51.5)	.13
Male	225 (53.1)	441 (48.5)	
Smoking status	391 (92.2)	850 (93.4)	.54
Follow-up time from initial diagnosis, median (IQR), mo	12.7 (8.7-17.7)	17.5 (12.1-24.0)	<.001
Time from initial diagnosis to treatment, median (95% CI), mo	1.3 (0.9-1.9)	2.7 (1.9-3.6)	<.001
Disease stage			
Stage II	192 (45.3)	524 (57.6)	<.001
Stage IIIA	232 (54.7)	386 (42.4)	
Histologic type			
Squamous	154 (36.3)	239 (26.3)	.001
Nonsquamous	266 (62.7)	663 (72.9)	
Unknown	4 (1.0)	8 (0.9)	
Practice type			
Academic	91 (21.5)	168 (18.5)	.22
Community	333 (78.5)	742 (81.5)	
Biomarker testing prior to CIO receipt			
* EGFR*	212 (50.0)	619 (68.0)	<.001
* ALK*	211 (49.8)	552 (60.7)	<.001
PD-L1	223 (52.6)	657 (72.2)	<.001
PD-L1 status			
<1%	69 (16.3)	104 (11.4)	<.001
1%-49%	86 (20.3)	324 (35.6)	
≥50%	68 (16.0)	229 (25.2)	
Not tested or unknown	201 (47.4)	253 (27.8)	
Metastatic disease			
Patients, No.	37	95	
Bone	11 (29.7)	26 (27.4)	.96
Adrenal	3 (8.1)	12 (12.6)	.67
Brain	12 (32.4)	34 (35.8)	.87
Liver	6 (16.2)	12 (12.6)	.80
Pleural	5 (13.5)	14 (14.7)	>.99

Abbreviations: CIO, chemoimmunotherapy; PD-L1, programmed cell death ligand 1.

In terms of smoking history, there was an equal proportion of smokers in both neoadjuvant and adjuvant cohorts (391 [92.2%] and 850 [93.4%]; *P* = .54). The median (IQR) follow-up time from initial diagnosis was significantly longer in the adjuvant cohort than the neoadjuvant cohort (17.5 [12.1-24.0] months vs 12.7 [8.7-17.7] months; *P* < .001). This was mirrored by longer median time from initial diagnosis to treatment in the adjuvant vs the neoadjuvant group (2.7 [95% CI, 1.9-3.6] months vs 1.3 [95% CI, 0.9-1.9] months; *P* < .001).

In terms of NSCLC stage, 716 patients (53.7%) were diagnosed with stage II and 618 patients (46.3%) were diagnosed with stage IIIA. Patients in the neoadjuvant cohort had a higher stage at diagnosis compared with patients in the adjuvant cohort (stage II: 192 [45.3%] vs 524 [57.6%], *P* < .001; stage IIIA: 232 [54.7%] vs 386 [42.4%], *P* < .001). Most patients had a nonsquamous histologic type (929 [69.6%]) followed by a squamous histologic type (393 [29.5%]), with 12 patients (0.9%) who did not have a specific histologic type. Patients who received neoadjuvant treatment were significantly more likely than those who received adjuvant treatment to have a squamous histologic type (154 [36.3%] vs 239 [26.3%]; *P* = .001).

**Figure 1.**
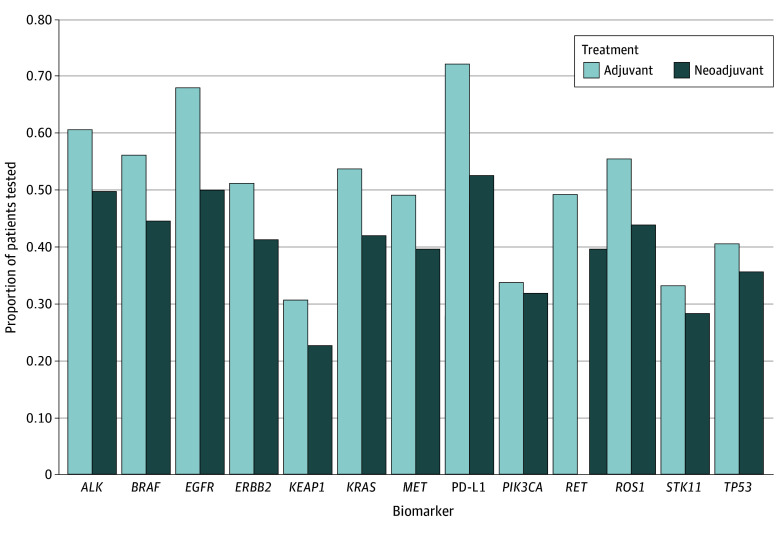
Biomarker Testing Rates by Neoadjuvant or Adjuvant Treatment PD-L1 indicates programmed cell death ligand 1.

Information on biomarker testing both from immunohistochemistry for PD-L1 status and genomic testing for actionable genomic alterations were available for a subset of patients (Figure 1). For example, 59.2% of patients (251) in the neoadjuvant cohort and 79.5% of patients (723) in the adjuvant cohort had available data on genomic biomarker testing. Biomarker testing rates were lower in the neoadjuvant than adjuvant cohort (*EGFR* alteration testing: 50.0% [212] vs 68.0% [619], *P* < .001; *ALK* alteration testing: 49.8% [211] vs 60.7% [552], *P* < .001). A total of 66.0% of patients (n = 880) had PD-L1 testing, with the distribution described in the Table. PD-L1 testing frequency prior to the start of therapy was lower in the neoadjuvant cohort compared with the adjuvant cohort (52.6% [223] vs 72.2% [657]; *P* < .001). Among patients with known PD-L1 results, 84.2% (553) in the adjuvant cohort had a PD-L1 threshold greater than 1% compared with 69.1% (154) in the neoadjuvant group.

Time-to-treatment analysis was conducted for both neoadjuvant and adjuvant cohorts. For the neoadjuvant cohort (n = 424), the median time from initial diagnosis to initiation of treatment was 1.3 (95% CI, 0.9-1.9) months. Subsequently, the median time from start of neoadjuvant treatment to surgical resection for the same cohort was 3.1 (95% CI, 3.02-3.22) months. For the adjuvant cohort (n = 910), the median time to initiation of treatment after surgical resection was 2.7 (95% CI, 1.9-3.6) months. The total duration of therapy for patients receiving adjuvant immunotherapy was distributed as follows: less than 3 months (145 [15.9%]), 3 to less than 6 months (175 [19.2%]), 6 to less than 12 months (323 [35.5%]), and 12 months or longer (267 [29.3%]). Kaplan-Meier survival analysis demonstrated significant differences in 18-month clinical DMFS related to the duration of adjuvant immunotherapy (*P* < .001). Specifically, shorter therapy durations were associated with the lowest survival probabilities (<3 months: 59.2%; 95% CI, 47.0%-74.5%), while longer durations were associated with the highest survival probabilities (≥12 months: 95.24%; 95% CI, 92.5%-98.1%), illustrating a possible dose-response association (eFigure 1 in Supplement 1).

**Figure 2.**
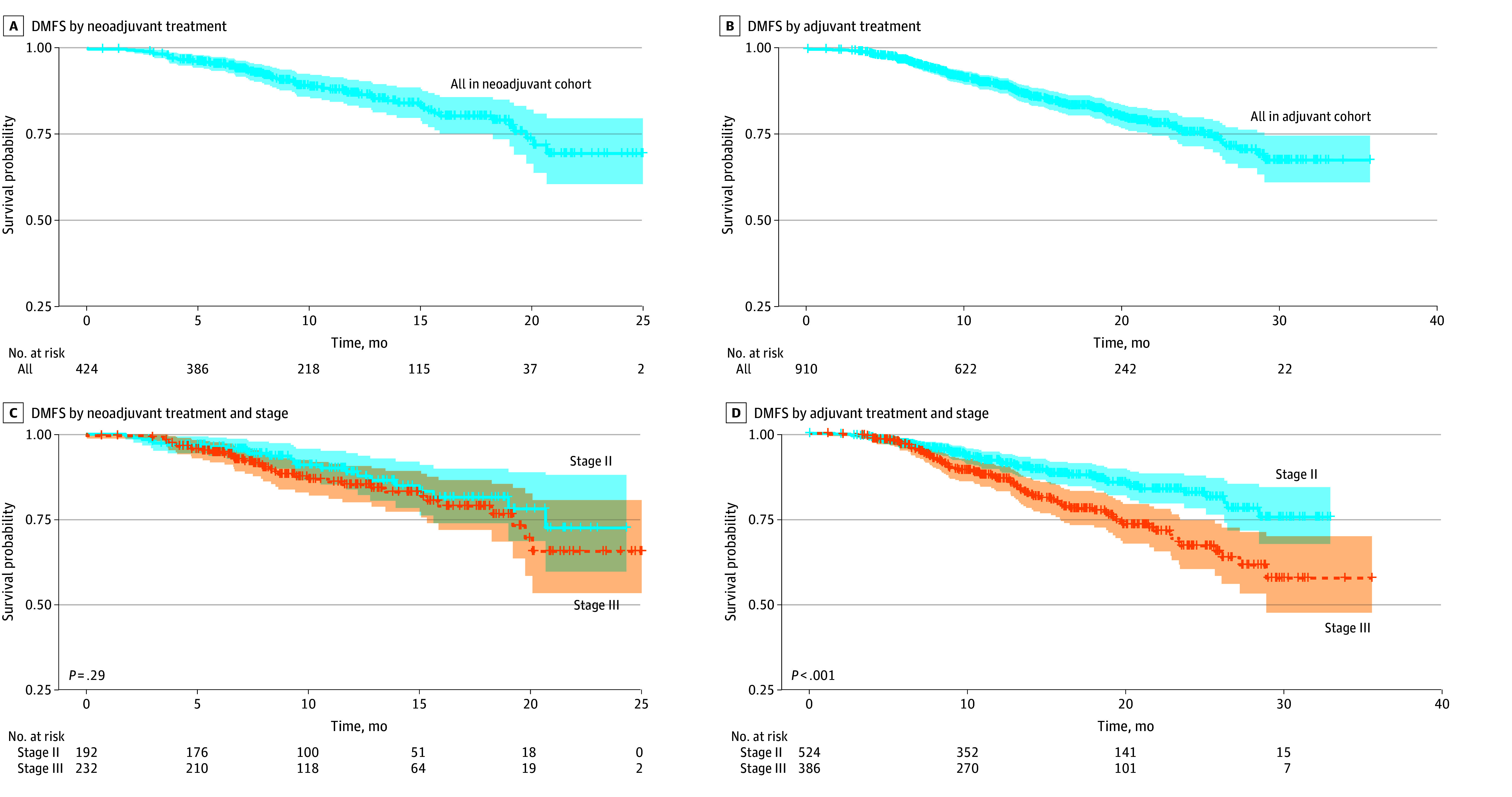
Clinical Distant Metastasis–Free Survival (DMFS) by Neoadjuvant or Adjuvant Treatment and Cancer Stage Shaded area indicates 95% CI.

**Figure 3.**
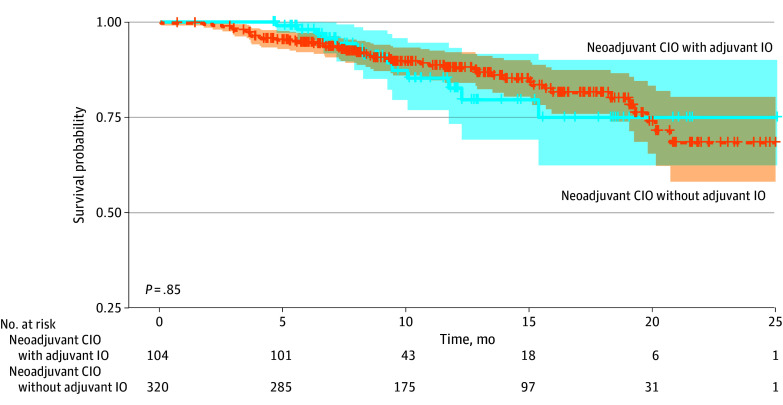
Sensitivity Analysis of Clinical Distant Metastasis–Free Survival (DMFS) Among Patients Receiving Neoadjuvant Chemoimmunotherapy (CIO) With or Without Subsequent Adjuvant Immunotherapy (IO) Shaded area indicates 95% CI.

The clinical DMFS at 18 months was 80.2% (95% CI, 75.0%-85.7%) for the neoadjuvant cohort compared with 83.0% (95% CI, 80.0%-86.0%) for the adjuvant cohort (Figure 2). Further subgroup analysis based on stage revealed an 18-month clinical DMFS of 81.5% (95% CI, 73.9%-89.9%) for stage II and 78.9% (95% CI, 72.0%-86.6%) for stage IIIA in the neoadjuvant setting, while the clinical DMFS was 87.3% (95% CI, 83.9%-90.9%) and 77.4% (95% CI, 72.4%-82.7%) for stage II and IIIA, respectively, in the adjuvant setting. The difference in clinical DMFS identified for the sensitivity analysis was not significant among the neoadjuvant chemoimmunotherapy group vs without subsequent adjuvant immunotherapy (74.8% [95% CI, 62.1%-90.0%] vs 81.3% [95% CI, 75.7%-87.3%]) (Figure 3). For patients who experienced distant metastases (n = 132), brain (46 [34.8%]), bone (37 [28.0%]), and pleura (19 [14.4%]) were the most common sites of metastases identified. The pattern of metastasis did not differ among the 2 cohorts (Table).

**Figure 4.**
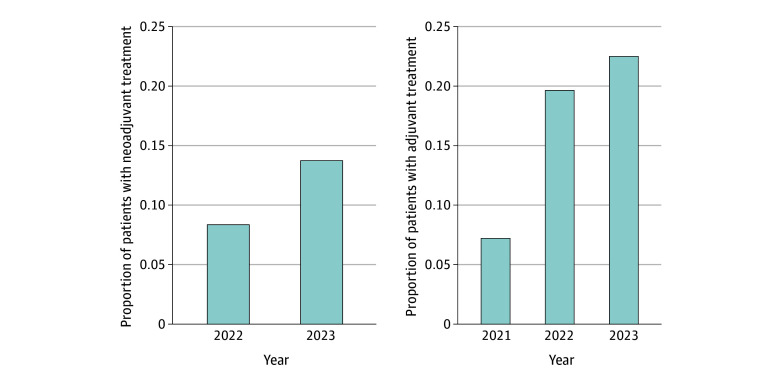
Neoadjuvant or Adjuvant Chemoimmunotherapy Use by Year

Further stratification based on PD-L1 did not identify any difference in clinical DMFS for the neoadjuvant cohort based on PD-L1 staining of less than 1%, 1% to 49%, and 50% or greater. For the adjuvant cohort, the clinical DMFS did not differ significantly based on PD-L1 expression. In the multivariate Cox proportional hazards regression model analysis for the adjuvant cohort, 1 clinical covariate that was significant was stage IIIA, with an HR of 2.20 (95% CI, 1.46-3.32). Time to first metastases did not differ based on smoking exposure or *KRAS* alteration status in both neoadjuvant and adjuvant settings (eFigure 2 in Supplement 1). Chemoimmunotherapy among patients who received surgery increased from 2022 to 2023: from 8.4% (82 of 966) to 13.8% (254 of 1842) in the neoadjuvant setting and from 19.7% (372 of 1887) to 22.6% (401 of 1776) in the adjuvant setting (Figure 4).

## Discussion

In this nationwide study of patients with resectable NSCLC, we found that chemoimmunotherapy use in the neoadjuvant and adjuvant settings had favorable clinical DMFS. Although uptake of chemoimmunotherapy drugs has increased since initial FDA approvals, of those patients who underwent surgery, fewer than 30% of eligible patients received chemoimmunotherapy in 2023, indicating that patterns of care, education, and potential barriers of adoption should be explored.

Despite guidelines and expert panel consensus recommendation for determination of *EGFR* and *ALK* alteration status for patients being considered for neoadjuvant or adjuvant systemic therapy,^14^ the present study identified that biomarker testing in both the neoadjuvant and adjuvant therapy cohort was approximately 50% to 60%. This finding suggests a need for improvement in incorporating biomarker testing in workflows for treatment of patients with resectable NSCLC. A recent survey on biomarker testing conducted by the International Association for the Study of Lung Cancer showed that key barriers to optimal biomarker testing are cost (27.2%), time (13.9%), tissue sample quality (13.8%), access (12.8%), and awareness (8.0%).^15^ Cost remains a substantial concern affected by complex reimbursement systems, with many institutions reporting only partial or no reimbursement for biomarker testing. The time required for testing delays treatment decisions, while insufficient tissue sample quality further compounds this problem, particularly in early-stage disease when tissue biopsies are often limited.

Despite these challenges, biomarker testing in early-stage NSCLC is vital given that patients with NSCLC with *EGFR* and *ALK* alterations either were excluded from studies incorporating neoadjuvant chemoimmunotherapy (eg, Checkmate 816 [A Neoadjuvant Study of Nivolumab Plus Ipilimumab or Nivolumab Plus Chemotherapy Versus Chemotherapy Alone in Early Stage Non-Small Cell Lung Cancer]^5^) or did not show any evidence of benefit from receiving immunotherapy in the adjuvant setting (ie, IMPower010 [Study to Assess Safety and Efficacy of Atezolizumab (MPDL3280A) Compared to Best Supportive Care Following Chemotherapy in Patients With Lung Cancer]^6^). Given that there is evidence of improved outcomes with targeted therapy in the adjuvant setting for these patients—as demonstrated by data from the ADAURA (AZD9291 Versus Placebo in Patients With Stage IB-IIIA Non-small Cell Lung Carcinoma, Following Complete Tumour Resection With or Without Adjuvant Chemotherapy) trial, using osimertinib in *EGFR*-mutant NSCLC,^16^ and the ALINA (A Study Comparing Adjuvant Alectinib Versus Adjuvant Platinum-Based Chemotherapy in Patients With ALK Positive Non-Small Cell Lung Cancer) trial, using alectinib in *ALK*-positive NSCLC,^17^ studies—biomarker testing is imperative. Furthermore, for patients with NSCLC with *EGFR* or *ALK* alterations, neoadjuvant chemoimmunotherapy or adjuvant immunotherapy is generally not recommended.

We defined neoadjuvant therapy as chemoimmunotherapy use preceding surgery. We found that most patients in the neoadjuvant cohort had stage IIIA disease. The 18-month clinical DMFS was numerically higher for patients with stage IIIA disease in the neoadjuvant cohort compared with the adjuvant cohort (78.9% vs 77.4%). A meta-analysis of randomized clinical trials demonstrated overall survival benefit of the neoadjuvant approach in patients with stage III NSCLC, revealing a statistically significant HR of 0.67 (95% CI, 0.53-0.85).^18^ Similar results have been reported for patients with stage II to IIIB NSCLC in a retrospective analysis of the National Cancer Database.^19^ Additionally, a recent consensus statement from the International Association for the Study of Lung Cancer indicated a strong preference for neoadjuvant chemoimmunotherapy compared with upfront surgery for medically operable patients with resectable clinical stage IIIA NSCLC.^20^ Regardless, these patients should be evaluated by a multidisciplinary team to devise an individualized treatment plan, ideally in a tumor board setting and consisting of surgeons, medical oncologists, radiation oncologists, pathologists, pulmonologists, radiologists, and supportive care staff.

In the adjuvant cohort, although biomarker testing was generally higher than in the neoadjuvant cohort, there is room for improvement, especially regarding *EGFR* and *ALK* testing. In terms of known PD-L1 status, most patients (84.2%) who received immunotherapy had PD-L1 greater than 1%. This finding is consistent with clinical trial data showing incremental benefit with higher PD-L1 expression in the adjuvant setting. Although FDA approval of pembrolizumab in this setting is unrelated to PD-L1 expression, consideration of adjuvant immunotherapy for PD-L1 less than 1% is generally discouraged by expert consensus recommendations given concerns about effectiveness, although it is incorporated in routine clinical guidelines.^17^

We found a higher proportion of patients with stage II NSCLC in the adjuvant cohort (57.6%) compared with the neoadjuvant cohort (45.3%). This finding may reflect the preference for upfront surgery for medically operable patients with technically resectable clinical stage II disease. In the Checkmate 816 trial, patients with stage II disease derived a lower magnitude of EFS benefit from the addition of nivolumab (HR, 0.87; 95% CI, 0.48-1.56). Similarly, perioperative strategies, including the KEYNOTE 671^8^ and AEGEAN^9^ trials, have not clearly demonstrated the benefit of adding immunotherapy for stage II disease given the lack of patient selection based on PDL-1 status due to the small sample size of these subgroups. Despite this lack of evidence, a meta-analysis revealed a statistical EFS benefit of immunotherapy for patients with stage II disease when all neoadjuvant and perioperative trials were pooled, with an HR of 0.71 (95% CI, 0.55-0.92).^16^ It is important to consider that in the VIOLET (Video Assisted Thoracoscopic Lobectomy Versus Conventional Open Lobectomy) study, 95% of patients were able to receive neoadjuvant therapy compared with only 50% of patients who received indicated adjuvant chemotherapy, demonstrating that the neoadjuvant approach may ensure the highest probability of patients receiving all potentially beneficial therapeutic options.^21^ In the subgroup analysis by stage, the 18-month clinical DMFS for patients with stage II NSCLC in the adjuvant cohort was 87.3%, which was notably higher than the 81.5% observed in the neoadjuvant cohort.

In this study, we observed patterns of metastatic sites associated with treatment failure and disease progression that were consistent across both treatment cohorts. The most common sites of metastases included the brain, bone, liver, and pleura. This study provides valuable insights into the patterns of failure with chemoimmunotherapy in early-stage settings, highlighting the need to explore novel therapeutic options that may expand drug penetration into the central nervous system and that may address these metastatic sites more effectively.

### Limitations

Given the retrospective nature of the study, its limitations included lack of data for certain variables and missingness in data obtained from the EHR. It is possible that there is a lag in mortality data collection in the Flatiron Health database, which may result in a minor underestimation of events. However, multiple data sources were used to collect mortality data in the Flatiron Health database, and the lag is expected to be minimal and not affect study conclusions.^13,22^ Patients may also be lost to follow-up if they seek treatment or care in practices outside of the Flatiron Health network, leading to potentially incomplete patient-level clinical information. Such limitations may affect the generalizability of the results.

Additionally, the study results may be impacted by unmeasured confounding due to unmeasured and/or unobserved clinical, demographic, and/or other characteristics in the EHR; unmeasured confounding might affect treatment and care received as well as clinical outcomes. Moreover, the relatively short follow-up period may not fully capture the changes in care patterns that often follow regulatory approvals, potentially affecting the long-term applicability of the findings. In particular, data on local recurrence, patient comorbidities, and molecular subtype distributions were not available in the dataset at the time of data analysis. Despite this lack of data, the recency and robust sample size of our data provide unique strengths, enabling us to evaluate the immediate implications of these evolving care practices for clinical DMFS and patterns of distant recurrence. Given the aggressive nature of NSCLC and its rapid disease progression, this study offers a timely and valuable snapshot of the role of treatment paradigms in outcomes.

## Conclusions

This nationwide retrospective cohort study demonstrated the benefits of chemoimmunotherapy in the neoadjuvant or adjuvant settings, including favorable clinical DMFS outcomes, for patients with resectable NSCLC. Despite the increased adoption of these therapies since their initial FDA approval, less than 30% of potentially eligible patients received them in 2023, signaling the need to address barriers to broader implementation. With chemoimmunotherapy now used in both neoadjuvant and adjuvant settings, it is important to understand the contribution of different therapies and risk stratification using novel technologies, such as circulating tumor DNA, with improved patient selection and outcomes. Furthermore, this study highlights the need for continued exploration of therapeutic options for common metastatic sites and the value of clinical data in evaluating new treatment paradigms and adapting to evolving clinical landscapes.

## Notes

Supplement 1. **eFigure 1.** 18-Month Distant Metastases or Death Free Survival (rw-DMFS) Stratified by Time on Adjuvant Immunotherapy **eFigure 2.** rw-DMFS Stratified by KRAS-Mutant Versus Non-KRAS Patients

Supplement 2.
Data Sharing Statement
