# Supplementary material for: Clinical Outcomes of Perioperative Immunotherapy in Resectable Non–Small Cell Lung Cancer
Source: JAMA Netw Open. 2025 Jun 30;8(6):e2517953. doi: 10.1001/jamanetworkopen.2025.17953 (PMC12210081; doi:10.1001/jamanetworkopen.2025.17953)
Supplement: Supplement 1. — eFigure 1. 18-Month Distant Metastases or Death Free Survival (rw-DMFS) Stratified by Time on Adjuvant Immunotherapy eFigure 2. rw-DMFS Stratified by KRAS-Mutant Versus Non-KRAS Patients [file jamanetwopen-e2517953-s001.pdf]

Supplemental Online Content

Desai A, Schwed K, Kalesinskas L, et al. Clinical outcomes of perioperative immunotherapy in resectable non–small cell lung cancer. *JAMA Netw Open*. 2025;8(6): e2517953. doi: 10.1001/jamanetworkopen.2025.17953

eFigure 1. 18-Month Distant Metastases or Death Free Survival (rw-DMFS) Stratified by Time on Adjuvant Immunotherapy

eFigure 2. rw-DMFS Stratified by KRAS-Mutant Versus Non-KRAS Patients

This supplemental material has been provided by the authors to give readers additional information about their work.

© 2025 Desai A et al. *JAMA Network Open*

Supplemental Figure 1: 18-month distant metastases or death free survival (rw-DMFS) stratified by time on adjuvant immunotherapy

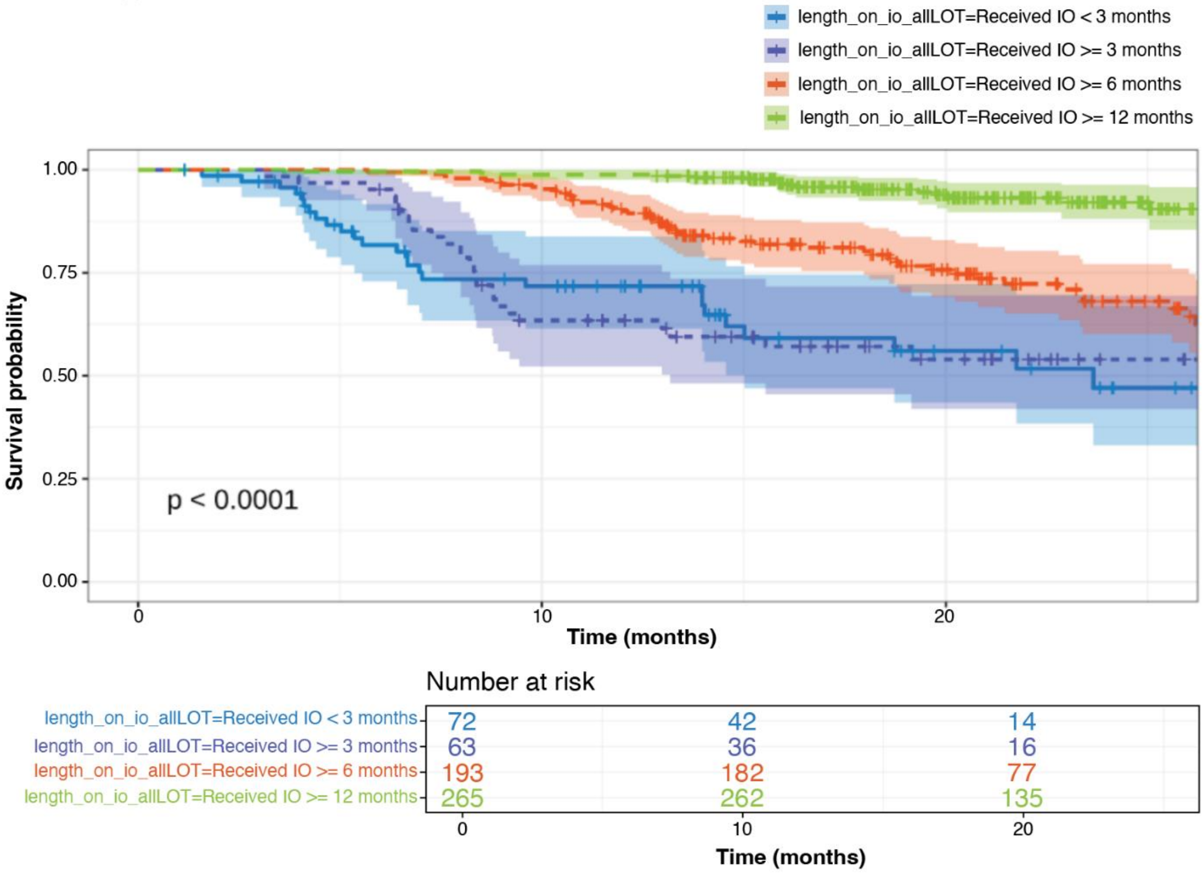

© 2025 Desai A et al. *JAMA Network Open*

Supplemental Figure 2: rw-DMFS stratified by KRAS-mutant versus non-KRAS patients

A) Neoadjuvant Cohort

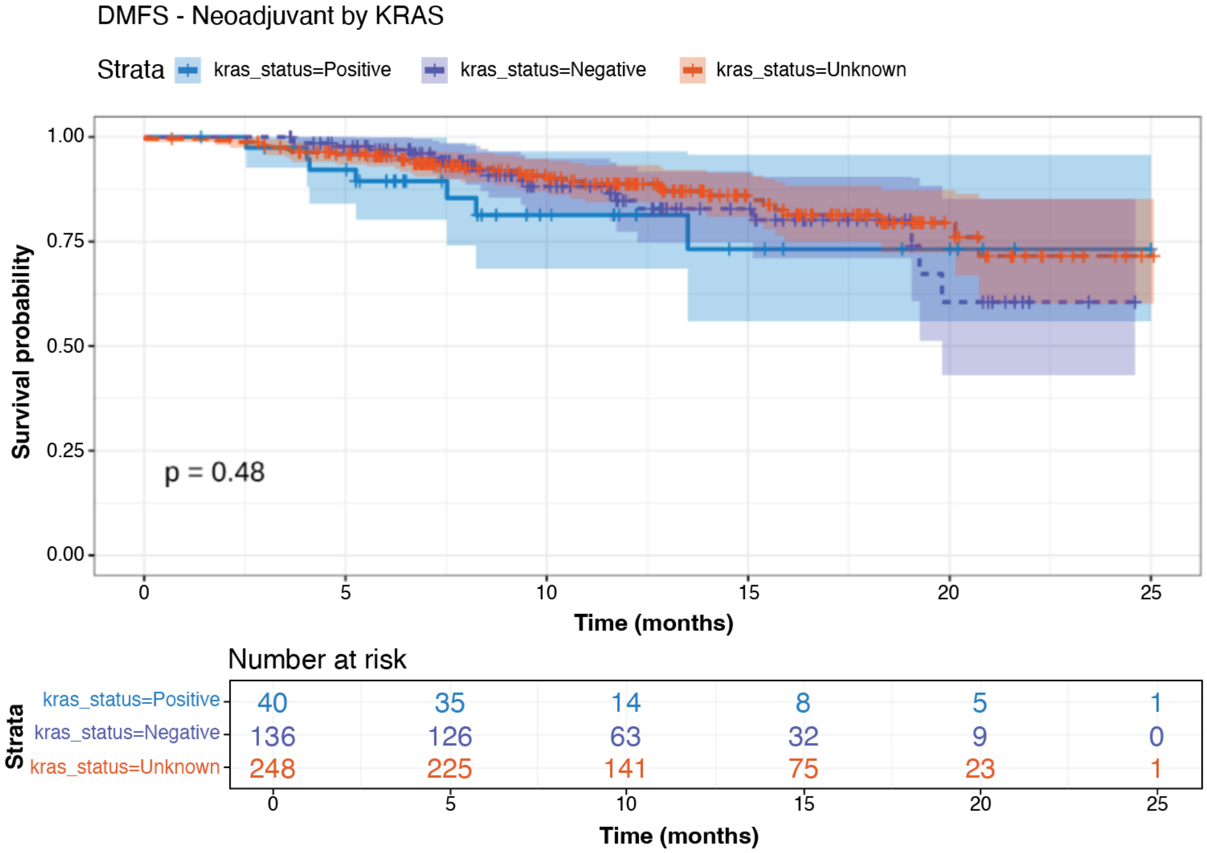

© 2025 Desai A et al. *JAMA Network Open*

B) Adjuvant Cohort

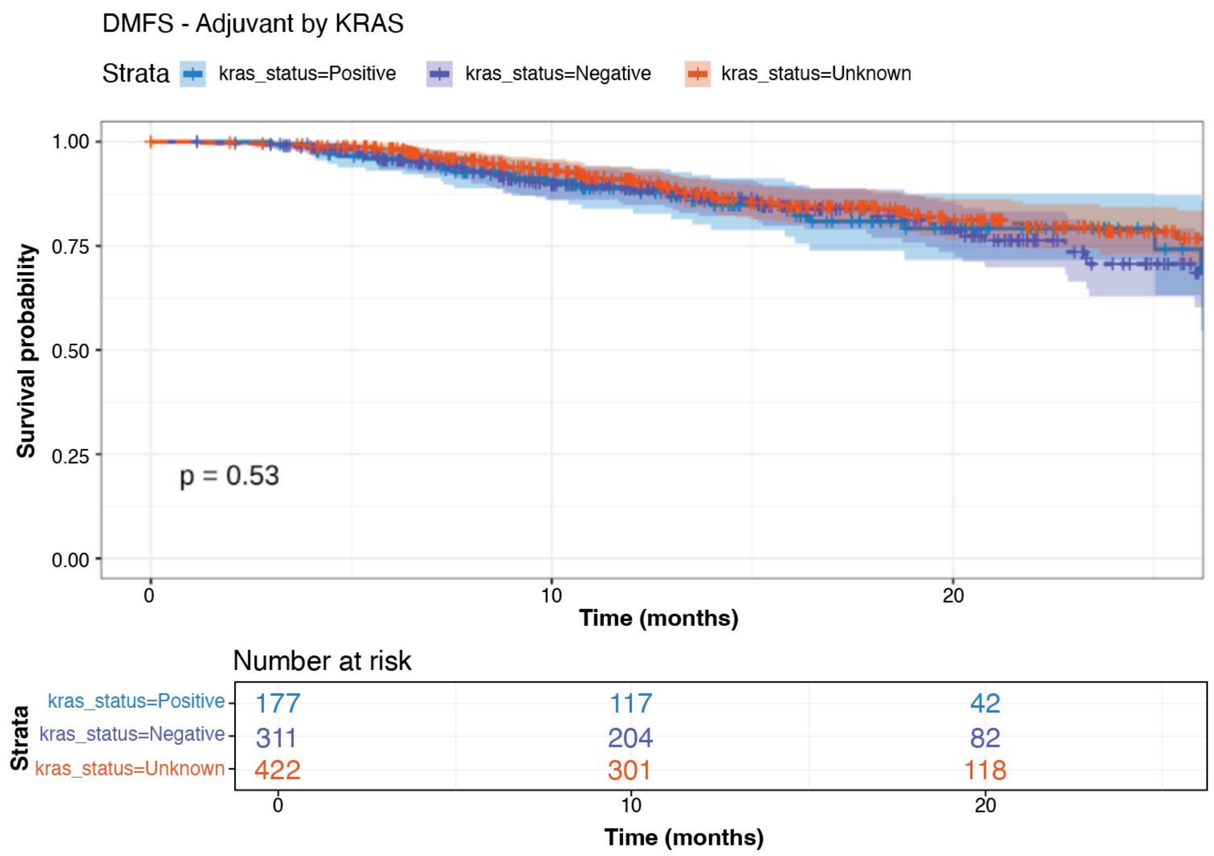

© 2025 Desai A et al. *JAMA Network Open*
