# Supplementary material for: Clinical Outcomes of Perioperative Immunotherapy in Resectable Non–Small Cell Lung Cancer
Source: JAMA Netw Open. 2025 Jun 30;8(6):e2517953. doi: 10.1001/jamanetworkopen.2025.17953 (PMC12210081; doi:10.1001/jamanetworkopen.2025.17953)
Supplement: Supplement 2. — Data Sharing Statement [file jamanetwopen-e2517953-s002.pdf]

# Data Sharing Statement

Desai. Clinical Outcomes of Perioperative Immunotherapy in Resectable Non–Small Cell Lung Cancer. *JAMA Netw Open*. Published June 30, 2025.
doi:10.1001/jamanetworkopen.2025.17953

**Data**
**Data available:** No
